# Endotoxin‐induced cerebral pathophysiology: differences between fetus and newborn

**DOI:** 10.14814/phy2.13973

**Published:** 2019-02-20

**Authors:** Susan Y. S. Feng, Jacob H. Hollis, Thilini Samarasinghe, David J. Phillips, Shripada Rao, Victor Y. H. Yu, Adrian M. Walker

**Affiliations:** ^1^ The Ritchie Centre Hudson Institute of Medical Research Clayton Victoria Australia; ^2^ Neonatal Directorate King Edward Memorial Hospital Perth Children's Hospital Subiaco Western Australia Australia; ^3^ Department of Physiology Monash University Clayton Victoria Australia; ^4^ Academic & Medical Portfolio Epworth HealthCare Richmond Victoria Australia; ^5^ Monash Newborn Monash Medical Centre Clayton Victoria Australia

**Keywords:** Brain, endotoxin, fetus, injury, newborn

## Abstract

As the comparative pathophysiology of perinatal infection in the fetus and newborn is uncertain, this study contrasted the cerebral effects of endotoxemia in conscious fetal sheep and newborn lambs. Responses to intravenous bacterial endotoxin (lipopolysaccharide, LPS) or normal saline were studied on three consecutive days in fetal sheep (LPS 1 *μ*g/kg, *n* = 5; normal saline *n* = 5) and newborn lambs (LPS 2 *μ*g/kg, *n* = 10; normal saline *n* = 5). Cerebro‐vascular function was assessed by monitoring cerebral blood flow (CBF) and cerebral vascular resistance (CVR) over 12 h each day, and inflammatory responses were assessed by plasma TNF alpha (TNF‐*α*), nitrate and nitrite concentrations. Brain injury was quantified by counting both resting and active macrophages in the caudate nucleus and periventricular white matter (PVWM). An acute cerebral vasoconstriction (within 1 h of LPS injection) occurred in both the fetus (ΔCVR +53%) and newborn (ΔCVR +63%); subsequently prolonged cerebral vasodilatation occurred in the fetus (ΔCVR −33%) in association with double plasma nitrate/nitrite concentrations, but not in the newborn. Abundant infiltration of activated macrophages was observed in both CN and PVWM at each age, with the extent being 2–3 times greater in the fetus (*P* < 0.001). In conclusion, while the fetus and newborn experience a similar acute disruption of the cerebral circulation after LPS, the fetus suffers a more prolonged circulatory disruption, a greater infiltration of activated macrophages, and an exaggerated susceptibility to brain injury.

## Introduction

Epidemiological data point to a causal relationship between infection and brain injury in the perinatal period (Dammann and Leviton 1998; Wu et al. 2003), with intrauterine infection strongly implicated in the pathogenesis of brain injury (Wu et al. 2003). Susceptibility appears not to be simply related to developmental maturity, as not only extremely preterm infants but also moderate and late‐preterm and term‐infants are vulnerable (Strunk et al. 2014). Thus, while preterm infants are at particularly high‐risk of infection‐induced cerebral palsy (CP) (Graham et al. 2004), more than half CP infants are born at term (Grether et al. 1992). Furthermore, postnatal infection has also recently been revealed as a causal factor of brain injury (Mitha et al. 2013; Hagberg et al. 2015).

We have demonstrated that not only the near‐term fetus, but also the term newborn lamb is susceptible to brain injury after endotoxin exposure (Feng et al. 2009, 2010). While some features of the circulatory and inflammatory responses of the fetus and newborn to LPS are similar, the underlying damaging mechanisms may well differ, and further study is required to identify the exact mechanisms and to assess whether the severity of infection‐related brain injury differs between fetal and newborn ages.

Cerebral hypoperfusion is a potential damaging mechanism as hypotension is commonly associated with infection in preterm neonates (Seri and Noori 2005) and in fetal animal models of endotoxemia (Garnier et al. 2001; Duncan et al. 2002; Dalitz et al. 2003; Peebles et al. 2003; Coumans et al. 2005). As the regulation of the cerebral circulation in conditions such as hypotension is different in the fetus and newborn (Volpe 2008), a critical difference in their relative circulatory responses might be anticipated. However, developmental differences in responses to other putatively damaging factors are less certain. Key factors implicated both in CBF regulation and causation of injury are proinflammatory cytokines, notably TNF‐*α*, and oxidative factors, such as nitric oxide (NO), both of which are neurotoxic (Dawson and Dawson 1996; Andrews et al. 1998; Haynes et al. 2009). Though both TNF‐*α* and NO products (plasma nitrates and nitrites) increase in both fetal and newborn lambs after endotoxin exposure (Feng et al. 2009, 2010), the relative changes are not clear.

Notwithstanding the developmental differences in susceptibility to infection, no direct comparisons have been made of the fetus and newborn in respect to the relative cerebral circulatory responses to endotoxaemia and potential underlying mechanisms. In this study we addressed these deficits by contrasting CBF, cytokine (TNF‐*α*) and oxidative (NO) responses of fetal and newborn lambs exposed to LPS. In respect of brain injury per se, a role for microglial activation in LPS induced‐periventricular white matter injury has been shown in fetal sheep (Mallard et al. 2003; Dean et al., 2009) and neonatal rabbit (Kannan, et al. 2011), but no quantitative comparison has been made between the fetus and the newborn in the same animal species. As microglia originate from primitive macrophages (Davoust et al. 2008; Ginhoux et al. 2010), which are a potential source of proinflammatory cytokines (Sawada et al. 1989; Lee et al. 1993) and NO (Moss and Bates 2001; Liu et al. 2002) as well as a key marker of neurotoxic injury (Kigerl et al. 2009; Feng et al. 2010), we also made quantitative comparisons of resting and activated macrophages at the two developmental stages.

## Methods

### Ethical approval

The experimental protocol was performed in accordance with guidelines established by the National Health and Medical Research Council of Australia and was approved by the Monash Medical Centre animal ethics committee at Monash University (approval no. MMCA/2003/43).

### Animal source and housing protocol

#### Fetal study

Ewes (Merino/Border‐Leicester cross) were brought into the Monash Medical Centre animal house 2 weeks before surgery. Ewes were fed ad libitum and, once feeding well, were surgically prepared for chronic study under general anesthesia.

#### Newborn study

Pregnant ewes were brought to the laboratory just prior to giving birth. After birth, the lambs were kept with the ewes for 1 day to drink colostrum so as to develop immunity to infection, then separated from the ewes, kept in company with other lambs and fed with commercial lamb milk replacer. The ewes were then returned to Monash University Animal Services. When gaining weight satisfactorily, the lambs were surgically prepared for study under general anesthesia. This was generally between 2 and 15 days.

### Surgical preparation

Surgical methods in preparation of fetal and newborn lambs have previously been described in full (Feng et al. 2009, 2010). For the fetal preparation, ewes (*n* = 10) were anesthetized at 123 ± 1 day gestation and both ewe and fetus were then instrumented for study. A nonocclusive double lumen catheter was inserted into the fetal carotid artery for arterial blood pressure (ABP) monitoring and blood sampling, and a nonocclusive single lumen catheter was inserted into the amniotic cavity for measuring amniotic pressure (PAF). A catheter was also positioned under the dura to record fetal intracranial pressure (ICP). Nonocclusive jugular venous catheters were inserted in both fetus and ewe for drug administration and blood sampling. To record fetal CBF, a transit‐time ultrasonic flow probe (2 mm diameter, Transonic Systems, Ithaca, NY) was positioned around the fetal superior sagittal sinus as previously described (Grant et al. 1995). At the completion of surgical procedures, both fetus and ewe were treated with antibiotics and analgesic. Similar procedures were followed in preparation of newborn lambs (*n* = 15) of age 12 ± 2 days. Animals were allowed 48 h postoperative recovery before being studied.

### Study conditions

**Figure 1:**
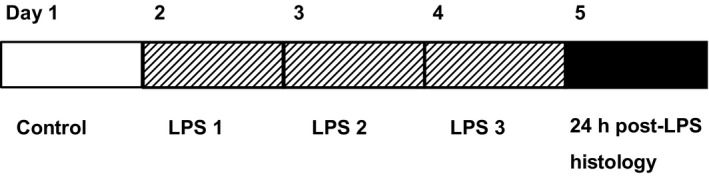
Summary of experimental timeline. Unanesthetized fetal sheep (LPS *n* = 5, NS *n* = 5) and newborn sheep (LPS *n* = 10, NS *n* = 5) were studied over a 5 day period. LPS was infused i.v. over 30 min on three consecutive days (1 *μ*g/kg fetus, 2 *μ*g/kg newborn). Brains were removed under anesthesia for histological analysis 24 h after the third LPS infusion.

Animals were studied over a 5 days period (Fig. 1): fetal studies began at a gestational age of 125 ± 1 days (mean ± SEM; 0.85 gestation, term at 147 days); newborn studies began at 12 ± 2 days. The flow probe was connected to the flowmeter (model T101 Ultrasonic Blood Flowmeter, Transonic Systems, Ithaca, NY). Vascular and intracranial catheters were connected to calibrated strain‐gauge manometers (Cobe CDX III, Cobe Laboratories; Lakewood, CO). Pressure and flow signals were low‐pass filtered at 100 Hz and recorded to hard disk (Powerlab, Chart v5.4.1, ADInstruments, Sydney, Australia.).

### Experimental protocol

#### Responses to LPS infusion

Lipopolysaccharide of 1 *μ*g/kg (*E*. *coli* O127‐B8, L3129, Sigma. USA) or normal saline was infused i.v. to fetuses by syringe pump (sp 100i infusion pump, WPI, USA) over 30 min on three consecutive days (LPS *n* = 5, NS *n* = 5). Fetal circulatory parameters including ABP, ICP, PAF and CBF were recorded continuously before and up to 12 h after LPS infusions; measurements were repeated 24 h post‐LPS. Blood samples were collected from fetuses for arterial blood gas, and the measurement of TNF‐*α* and total nitrate/nitrite at 0, 1, 2, 4, 6, 8, 10, 12, and 24 h post‐LPS infusion.

A similar experimental protocol was followed in newborn lambs (LPS *n* = 10, NS *n* = 5), except that the LPS dosage differed. In preliminary studies, we found that the newborn lamb tolerated a dose of 2 *μ*g/kg with zero mortality (Feng et al. 2010). Fetuses given LPS at 2 *μ*g/kg did not survive the 5 day period of study, whereas at a lower dose (1 *μ*g/kg) 5 of 9 fetuses survived (Feng et al. 2009).

#### Histology

At the conclusion of physiological studies, brains were paraffin embedded after perfusion fixation with 2 L of heparinized saline (10 IU/mL) followed by 2 L of 4% paraformaldehyde in 0.1 mol/L phosphate buffer‐saline (pH 7.4) (Feng et al. 2009, 2010). We selected regions topographically using the published nomenclature of sections 800–1000 for the sheep brain (Johnson et al. 1994). These regions include periventricular white matter (PVWM) and the caudate nucleus (CN) (Nagareddy et al. 2009), both of which have been shown to be damaged by LPS (Mallard et al. 2003; Hutton et al. 2008). Paraffin blocks were sectioned at 10 *μ*m, and stained with hematoxylin and eosin. The number of resting and active macrophages within the CN and PVWM was determined within one field of view (200× magnification) from each of three separate tissue sections. Resting macrophages were defined as mononuclear and agranular, whereas active macrophages were defined as multinuclear and granular (Feng et al. 2010). For each animal, the numbers of resting and active macrophages from each section and within each brain region were summed prior to statistical analysis.

### Data analysis and statistics

Data were analyzed using one‐way (physiological data) or two‐way (histology data) repeated measures ANOVA; when data failed normality and equal variance tests, Kruskal–Wallis one‐way ANOVA was applied. The source of differences detected by ANOVA was identified using Student‐Newman‐Keuls or Dunn's posthoc analysis. Data are presented as mean ± SEM. Quantitative histology comparisons were made by use of two‐way ANOVA with one repeated measure and a Student–Newman–Keuls posthoc test. All tests employed SigmaPlot v12.0 (SPSS, http://www.spss.com) and *P* < 0.05 was considered to be significant.

## Results

### Brain histology and morphometry

**Figure 2:**
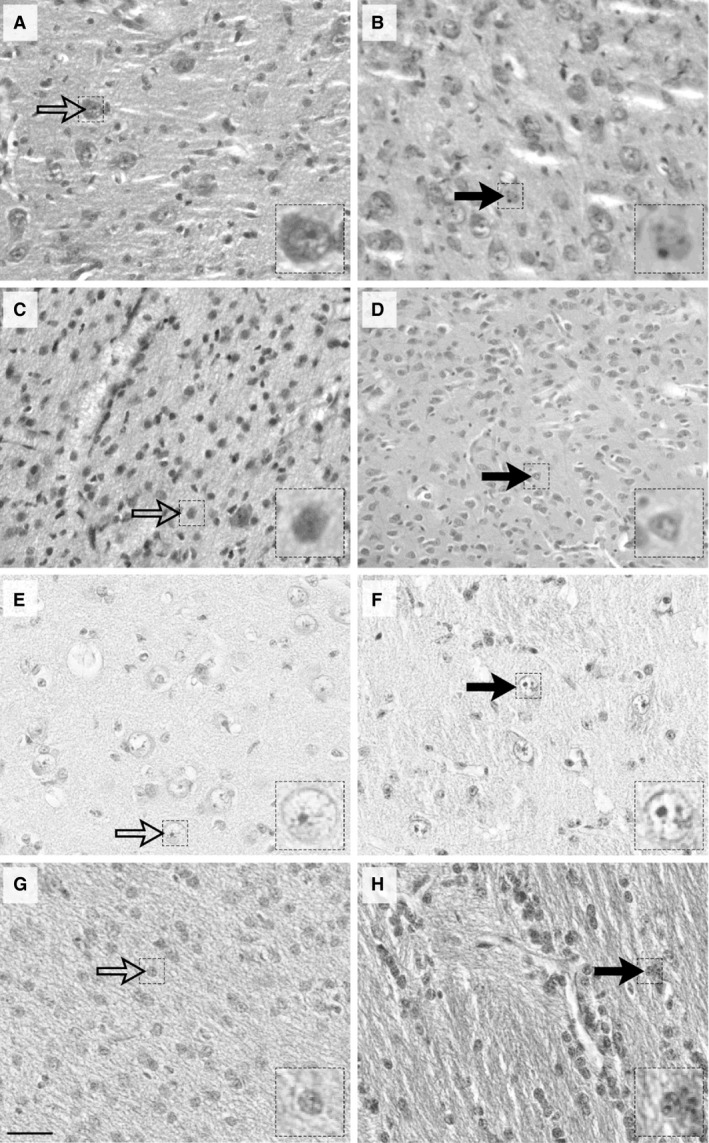
Photomicrographs depicting the effects of i.v. saline (left column) or LPS (right column) on the number of resting and activated macrophages in the caudate nucleus (A,B,E,F) and periventricular white matter of the thalamus (C,D,G,H) from fetal (A–D) or newborn (E–H) lambs. Open arrows identify resting macrophages which are mononuclear and agranular, while closed arrows identify activated macrophages which are multinuclear and granular. Note the robust increase of activated macrophages following LPS treatment, particularly in fetal lambs. Scale bar: 50 *μ*m; high magnification insert, 15 *μ*m.

Morphological effects of i.v. saline or LPS infusions on the number of resting and activated macrophages in the CN and PVWM of the thalamus of fetal or newborn lambs are illustrated in Figure 2. Activated macrophages were increased significantly following LPS treatment, particularly in fetal lambs.

Results of quantitative morphometric analyses of macrophage numbers in fetal and newborn sheep are detailed in Table 1.

**Table 1:** Macrophage counts (mean ± SEM) in caudate nucleus (CN) and periventricular white matter (PVWM) of LPS‐treated and saline‐treated fetal and newborn lambs

Lambs	Region	Macrophages (cells/field)	LPS (*n* = 5)	Saline (*n* = 5)	*P‐*value LPS versus saline
		Resting	10 ± 1	9 ± 3	NS
	CN	Active	100 ± 7	37 ± 7	<0.001
Fetus		*P‐*value Resting versus active	<0.001	<0.05	
		Resting	10 ± 2	6 ± 2	NS
	PVWM	Active	117 ± 3	45 ± 8	<0.001
		*P‐*value Resting versus active	<0.001	<0.001	
		Resting	13 ± 3	8 ± 1	NS
	CN	Active	34 ± 2	15 ± 2	<0.001
Newborn		*P‐*value Resting versus active	<0.001	NS	
		Resting	15 ± 2	11 ± 1	NS
	PVWM	Active	29 ± 4	15 ± 1	<0.001
		*P‐*value Resting versus active	<0.005	NS	

In LPS‐treated fetal sheep, activated macrophages significantly exceeded resting macrophages by 10‐fold in both CN and PVWM in LPS‐treated groups (*P* < 0.001, ANOVA). In saline‐treated control fetuses, activated macrophages also exceeded resting macrophages, but the extent of the difference was less, being fourfold greater in CN and eightfold greater in PVWM (*P* < 0.05 and *P* < 0.001 respectively, ANOVA, Table 1). Comparing LPS and saline‐treated fetal sheep, activated macrophages in the LPS‐treated group were three times greater than in the group treated with saline, in both brain regions (*P* < 0.001, ANOVA); by contrast, resting macrophage were not different between LPS and saline treatments.

In LPS‐treated newborn lambs, activated macrophages were twofold greater than resting macrophages in both CN and PVWM (*P* < 0.001 and *P* < 0.005 respectively, ANOVA, Table 1). In saline‐treated newborn lambs, by contrast to the fetal lambs, there was no resting‐active macrophage difference in either brain region. Comparing LPS and saline‐treated newborn sheep, activated macrophages in the LPS‐treated group were twice those in the saline‐treated group, in both brain regions (*P* < 0.001, ANOVA), whereas resting macrophages, as in fetal sheep, did not differ between treatments.

**Figure 3:**
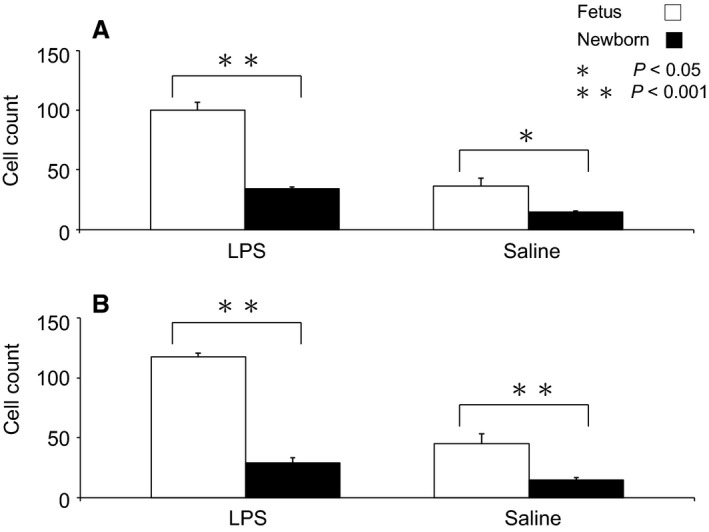
Activated macrophage cell counts (cells per field) contrasted in fetal and newborn lambs treated with LPS or saline (*n* = 5 in each group). Panel A: in CN; Panel B: in PVWM. Note the much greater fetal‐newborn difference after LPS treatment.

Comparison of activated macrophage counts in the fetus and newborn are illustrated in Figure 3. The numbers of activated macrophages were substantially greater in the fetus, being approximately three to fourfold greater than in the newborn in both brain regions in LPS‐treated groups (*P* < 0.001, ANOVA) and two to threefold greater in the saline‐treated groups (*P* < 0.05 in CN and *P* < 0.001 in PVWM, Fig. 2). Resting macrophage numbers were similar in fetus and newborn LPS (10 ± 1 vs. 13 ± 3 cells/field in CN, *P* > 0.05, ANOVA; 10 ± 2 vs. 15 ± 2 cells/field in PVWM, *P* > 0.05, ANOVA) and saline‐treated lambs (9 ± 3 vs. 8 ± 1 cells/field in CN, *P* > 0.05, ANOVA; 6 ± 2 vs. 11 ± 1 cells/field in PVWM, *P* > 0.05, ANOVA).

### Cerebral hemodynamics

**Figure 4:**
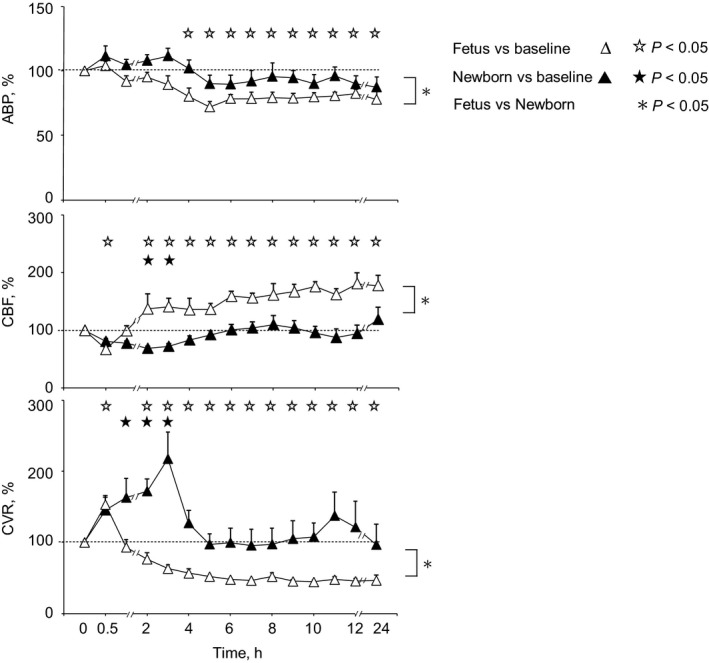
Arterial blood pressure (ABP), cerebral blood flow (CBF), and cerebral vascular resistance (CVR) responses to the first LPS treatment in fetuses (Δ, *n* = 5) and newborn lambs (▲, *n* = 10). Symbols denote significant differences (*P* < 0.05) versus baseline (time zero) for fetus (Δ) and newborn (▲), and for fetus versus newborn (*). Fetal ABP fell 4 h after LPS treatment and remained depressed at 24 h, whereas ABP remained unchanged in the newborn. Fetal CBF had a biphasic response to LPS, with an initial decrease followed by a prolonged increase for 24 h, whereas in newborn CBF there was only a brief decrease. Fetal CVR had a biphasic response to LPS, with an initial cerebro‐vasoconstriction followed by a prolonged cerebro‐vasodilation for 24 h, whereas there was only a brief cerebro‐vasoconstriction in the newborn.

Baseline (Pre‐LPS) values of ABP, CBF, and CVR in fetal and newborn groups are shown in Table 2. The significant difference in ABP and CBF are as expected in healthy fetal and newborn lambs (Purves and James 1969; Grant et al. 1995). Following the first LPS (LPS1) infusion, biphasic response patterns in CBF and CVR occurred in both the fetus and newborn, each characterized by an initial vasoconstriction, and then by subsequent vasodilatation (Fig. 4). However, the pattern differed between fetus and newborn (*P* < 0.001, Kruskal‐Wallis) in that CVR fell below baseline, causing CBF to rise significantly in the fetus, but not in the newborn. Additionally, an earlier onset of vasodilatation in the fetus caused CVR to quickly recover to baseline at 1 h, whereas in the newborn group it took 4 h for the early vasoconstriction to resolve. ABP was unchanged throughout the early phase of the response in both groups, but subsequently the fetus experienced a persistent hypotension in a pattern which differed significantly from the newborn (*P* < 0.001, Kruskal–Wallis), in which the ABP did not fall. Overall, similar patterns of circulatory disruption were evident following the second (LPS2) and third (LPS3) infusions (data not shown). No major arterial blood gas or pH differences pre‐ and post‐LPS were evident compared to the control groups (Feng et al. 2009, 2010).

**Table 2:** Baseline (pre‐LPS) values of ABP, CBF, CVR, nitrate/nitrite and TNF‐*α* in the fetus and newborn (mean ± SEM). NS, not significant. Other abbreviations defined in Methods

	Fetus (*n* = 5)	Newborn (*n* = 5)	*P‐*value fetus versus Newborn
ABP, mmHg	50.8 ± 2.4	82.9 ± 3.7	<0.001
CBF, mL/min	8.9 ± 0.7	16.6 ± 1.2	<0.001
CVR, mmHg·mL^−1^·min	4.4 ± 0.5	3.1 ± 0.4	NS
TNF‐*α*, ng/mL	0.13 ± 0	0.13 ± 0	NS
Nitrate/Nitrite (*μ*mol/L)	27.5 ± 4.5	4.7 ± 0.5	0.008

### Plasma cytokines and nitrate/nitrite

**Figure 5:**
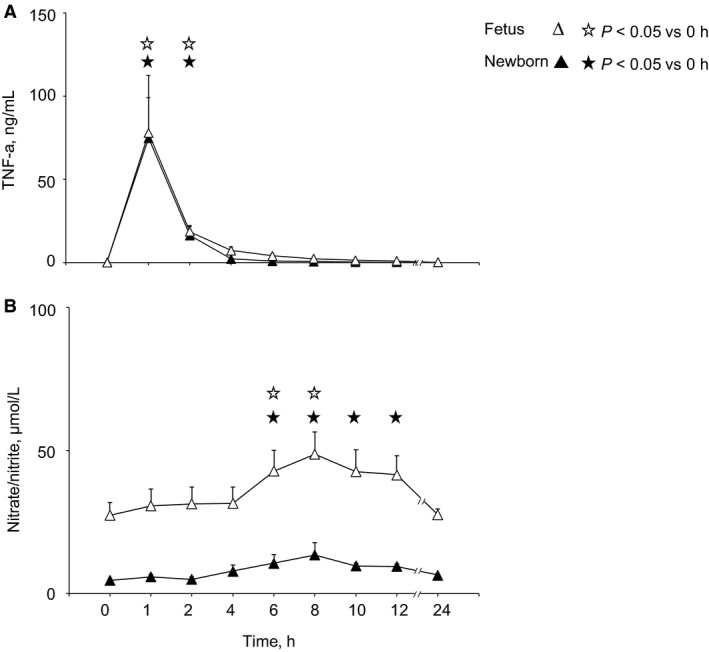
Plasma TNF‐*α* (A) and nitrate/nitrite (B) concentrations in fetus (Δ, *n* = 5) and newborn (▲, *n* = 5) after LPS. TNF‐*α* responses were the same in the fetus and newborn whereas baseline and peak nitrate/nitrite concentrations after LPS treatment were higher in the fetus than in newborn. Symbols denote significant differences (*P* < 0.05) versus baseline (time zero) for fetus (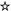) and newborn (★)

Baseline (pre‐LPS) values of plasma TNF‐*α* and nitrate/nitrite in fetal and newborn LPS treatment groups are shown in Table 2. After LPS infusion, we observed a similar pattern of TNF‐*α* response in the fetus and newborn (Fig. 5A). In each group, plasma TNF‐*α* levels (mean ± SE, ng/mL) were increased at essentially the same time of ~1 h after LPS and to the same degree (78 ± 35 in the fetus and 75 ± 24 in the newborn).

Substantially different increases in plasma nitrate/nitrite occurred in the fetus and newborn (Fig. 5B, *P* < 0.001, ANOVA). Though the pattern of change was similar, with values reaching approximately twice the baseline level occurring 6–8 h post‐LPS, the extent of the increase was twofold greater in the fetus (plasma nitrate/nitrite 28 ± 5 *μ*mol/L pre‐LPS, 43 ± 7 *μ*mol/L post‐LPS) than in the newborn (plasma nitrate/nitrite 5 ± 1 *μ*mol/L pre‐LPS, 11 ± 3 *μ*mol/L post‐LPS).

## Discussion

### Summary of findings

Perinatal brain injury arising from infection is a major clinical problem but, while significant differences exist in the susceptibility of fetus and newborn to injury, a developmental comparison of potential mechanisms has not been made. This study in fetal and newborn lambs represents the first experimental comparison of hemodynamic and inflammatory factors as potential causes of brain injury after LPS injection. The primary hemodynamic difference lies in the ability of the fetus to increase cerebral blood flow after LPS by prolonged cerebro‐vasodilation after a short‐lived (1 h) vasoconstriction; by contrast in the newborn cerebro‐vasoconstriction persists for 6 h in the absence of cerebral hypoxia (Feng et al. 2009, 2010). In keeping with the hemodynamic differences, fetal production of vasodilator nitric oxide (as indicated by plasma nitrate/nitrite) is significantly elevated compared to the neonate. Though the inflammatory response of the fetus and newborn appears similar (as indicated by plasma TNF‐*α*), the fetus has greater numbers of activated macrophages, indicative of greater cerebral injury after LPS injection. As activated macrophages are a significant source of high‐output iNOS that is potentially injurious in the brain (Hibbs et al. 1988), the larger activated macrophage response of the fetus and higher baseline circulating nitrate/nitrite level suggests that the near‐term fetal brain remains more vulnerable to injury than the newborn brain in the setting of infection. Thus, after LPS injection, the fetus appears more prone to cerebral injury than the newborn because of a greater vulnerability to inflammation, not because of cerebral hypoxia.

### Brain injury

We found evidence of LPS‐induced brain injury in the CN and the PVWM of both fetus and newborn. Neurons in all five LPS‐treated fetal brains exhibited an irregular cytoplasm lacking any definitive border, indicative of dying cells (Feng et al. 2009). A similar pattern of injury was found in four of five newborns (Feng et al. 2010). Quantitatively, macrophage numbers were significantly elevated by LPS injection in both the fetus and newborn, but the extent of the increase was much greater in the fetus (threefold) than in the newborn (twofold). Moreover, macrophages are more abundant in saline‐treated fetuses [(Feng et al. 2010) and Tables 1 and 2]; consequently, macrophage numbers were significantly greater in the fetus before and (more substantially) after LPS.

Microglial cells, as a unique type of tissue‐resident macrophage in the brain (Davoust et al. 2008) play an important role in the development of the inflammatory response in the developing brain (Chew et al. 2006; Kaur et al. 2007). Macrophages were found to have entered the nervous system by mid‐fetal life, and well‐differentiated microglia are present in both grey and white matter from 35 weeks’ gestation in human fetuses (Esiri et al. 1991). Though there is no previous quantitative comparison of the phenotypic expression of microglial cells in the developing brain of the same species (Guillemin and Brew 2004) the lower number of macrophages that we observed in the saline‐treated newborn compared with that in the fetus (Fig. 2) is in keeping with their observed disappearance from the cerebral parenchyma after birth in mice (Matsumoto and Ikuta 1985). In addition to the higher basal macrophage numbers in the fetal brain, the significantly greater increase in macrophage cell counts in the fetal than the newborn brain after LPS in our study (Fig. 2) suggests that the fetal brain is more susceptible to peripheral LPS than the newborn. This is consistent with the finding that pregnancy itself enhanced the inflammatory response of the cerebral circulation to endotoxin in rats (Cipolla et al. 2011). As the permeability of the blood–brain barrier increases after exposure to bacterial cell wall components (Rivest et al. 2000; Strunk et al. 2014), the greater inflammatory response in the fetus might also be related to the immaturity or disruption of the fetal blood–brain barrier (Guillemin and Brew 2004), or dysfunction of amoeboid microglial cells, the barrier which is believed to provide a necessary protection when the blood–brain barrier is deficient during the early developmental period (Kaur et al. 2007).

Brain lesions occurred in the fetus and newborn though there was no apparent deficit in cerebral oxygenation at either age. In the fetus, hypoxia was well compensated by a CBF increase that sustained oxygen transport (Feng et al. 2010). In the newborn, there was a period of hypoperfusion, though increased fractional oxygen extraction is likely to have preserved cerebral oxygenation and cerebral metabolic rate (Feng et al. 2009). Thus, in the short term, no cerebral hypoxia was likely after LPS.

Therefore, endotoxin‐induced perinatal brain injury can occur independently, without cerebral hypoperfusion or cerebral hypoxia, in the near‐term fetus and newborn.

### Cerebral hemodynamics

We observed a CBF reduction and a CVR increase within 1 h of LPS injection in the fetus and newborn (Fig. 3), consistent with previous studies showing the prompt appearance of cytokines in the brain within 1 h of LPS administration (Cai et al. 2000). The similarity of the timing in the fetus and newborn suggests a common mechanism in the initial inflammatory process, which is possibly cytokine‐induced cerebral vasoconstriction, as TNF‐*α* is a powerful vasoconstrictor in the adult brain (Giardina et al. 2002; Vecchione et al. 2009).

### Plasma cytokines

We observed a similar pattern of TNF‐*α* response in the fetus and newborn, suggesting both share the same proinflammatory pathway during the acute phase of endotoxemia. Plasma TNF‐*α* levels were increased at essentially the same time of ~1 h after LPS and to the same degree at both ages (to 78 ± 35 [mean ± SE] ng/mL in the fetus and to 75 ± 24 ng/mL in the newborn). Thus, over the developmental period of 0.85 gestation to 2 weeks postnatal age, the cytokine component of the immune response to endotoxemia appears to be unchanged in the lamb.

### Plasma nitrate/nitrite

Our studies demonstrated prolonged increases in CBF and decreases in CVR persisting from ~6 h onwards in fetus, but not in the newborn. Notably, vasodilatation occurs despite a loss of endothelium‐dependent (presumably endothelial nitric oxide synthase (eNOS) related) vasodilatation (Feng et al. 2009, 2010). The pattern of CVR reduction correlates with the time when the nitrate/nitrite level was increased in fetus, and is consistent with the timing of inducible nitric oxide synthase (iNOS) induction shown at ~5 h after LPS exposure (Lin et al. 2006). Thus, it appears that iNOS, not eNOS, may be the source of vasodilatory responses. Comparatively, the newborn does not experience the large magnitude nitrate/nitrite increment which is seen in the fetus (Fig. 4B), consistent with the study of Yang et al. (1996) showing the high capability for NO production in the fetus.

Other molecular mechanisms potentially underlying the differences in inflammation and vascular resistance between fetus and newborn could be initiated by LPS‐induced Nuclear Factor‐JB activation in fetal brain (Wang et al. 2007a). This molecule has widespread actions, releasing a number of cytokines and chemokines involved with the inflammatory signal to the central nervous system (Rivest 2003; Malaeb and Dammann 2009).”

### Perspectives and significance

Though hyperperfusion has been elicited in our fetal LPS model of global CBF measurement, but not in the newborn, CBF differs between brain regions and we cannot presume that the thalamus experiences a similar CBF response to that seen globally. For example, the white matter of the brain has a low‐energy requirement and may receive a blood flow as low as 20 mL/100 gm tissue/min, whereas other areas have high‐metabolic activity and require flow as high as 80 mL/100 gm tissue/min (Purves and James 1969; Jankowski 1982). Therefore, further detailed exploration on the endotoxin‐induced blood flow regionally within the brain is needed. These regional blood flow measurements would be complemented by evaluation of colocalized factors that may be involved in vasoconstriction‐induced tissue injury, with TNF‐*α* being a focus.

Notably, in an evaluation of the regional correlation of CBF with histological injury in experimental hypoxic‐ischemic encephalopathy, Back has questioned the long‐standing concept that hypoperfusion per se is the sole basis of injury (Back et al. 2006, 2007). It is supported by our findings that endotoxin‐induced brain injury with no hypoxic‐ischemic insult was associated with combined cerebral hypoperfusion and hyperperfusion in the fetuses, and with hypoperfusion followed by normal perfusion in the newborn (Feng et al. 2009, 2010). Furthermore, our data indicate that cerebral hypoperfusion is independent from hypotension in LPS‐induced fetuses and newborns.

After LPS exposure, the fetuses had a higher amount of NO and more activated macrophages production compared to the newborn lambs, suggesting the fetus is more vulnerable to an inflammatory‐induced brain injury which is independent of any hypoxic‐ischemic insult. This could be associated with the immaturity (Guillemin and Brew 2004) or deficiency of the fetal blood–brain barrier in the early developmental period (Kaur et al. 2007). Fetal vulnerability could also be due to the fact that pregnancy itself is a state of heightened inflammatory sensitivity. Thus, future study on NO suppression of fetal inflammation may help in treating infection antenatally.

Though cerebral tissue hypoxia was unlikely to be a factor in the injury seen in this study, infection could nevertheless add to the vulnerability of the fetus to hypoxic‐ischemic insult and exacerbate injury (Wang et al. 2007a). A comparative study combining infection with hypoxic‐ischemic insult in fetus and newborn would add clarity on the heightened risk of brain injury in fetal life. Our studies suggest that LPS‐induced brain injury is related to an initial rise of TNF‐*α*, followed by rise of NO and activated macrophages production, predominantly in the fetus. TNF‐*α* is one of the key proinflammatory cytokines, and outstanding in that it has a particularly rapid response to infection in ewes (Jones et al. 2004). To date, TNF‐*α* is the only proinflammatory cytokine which has a cytokine immunoassay validated specifically for sheep (Dr D Philips, personal communication) (Feng et al. 2009, 2010). At this time the relative time course and extent of TNF‐*α* changes are known for the adult, but are not entirely clear for the fetus and newborn, and so require further investigation. As TNF‐*α* is putatively responsible for the early vasoconstriction in our study, further study of its role in the long‐term sensitization to hypoxic‐ischemic injury would be of interest, as the mechanistic basis for sensitization is unknown. Such assessments could be achieved using a TNF‐*α* antagonist (Jones et al. 2004), testing the hypothesis that the cerebral vasoconstriction response would be attenuated, along with the extent of injury.

Because macrophages are a potential source of TNF‐*α* (Sawada et al. 1989; Lee et al. 1993) and NO (Moss and Bates 2001; Liu et al. 2002), as well as a key marker of neurotoxic injury (Kigerl et al. 2009; Feng et al. 2010; Anderson et al. 2015; Michels et al. 2015), further studies inhibiting NO, and suppressing the activated macrophages in the endotoxin‐induced fetal brain might shed light on strategies for perinatal neuroprotection.

Further study using immunoblotting, immunofluorescence, IHC, and real‐time PCR may help to determine other potential mechanisms causing circulatory disruption/injury, which could then be related to the extent of macrophage damage determined with macrophage morphometry.

Finally, these LPS‐injured animal models could be employed to investigate new therapy directed at accelerating the natural process of reendothelialization (for example using carbamylated‐recombinant human erythropoietin) (Coleman et al. 2006; Mennini et al. 2006; d'Uscio et al. 2007; Wang et al. 2007b).

## Conflict of Interest

None declared.
